# The effect of lavender aroma for anxiety disorder: a study protocol for a multicenter, double-masked, randomized, placebo-controlled clinical trial

**DOI:** 10.1186/s12906-023-04231-1

**Published:** 2023-11-06

**Authors:** Haruka Amitani, Ryusei Nishi, Kenichiro Sagiyama, Takamasa Fukumoto, Kouta Funakoshi, Naomi Takayanagi, Hiroko Watanabe, Masayuki Hirose, Koshiro Tagawa, Keiko Ota, Yoichi M. Ito, Akihiro Asakawa

**Affiliations:** 1 Department of Psychosomatic Internal Medicine, Kagoshima University Graduate School of Medical and Dental Sciences, Kagoshima, Japan; 2 Center for Clinical and Translational Research, Kyushu University Hospital, Fukuoka, Japan; 3 Center for Clinical Research and Innovation, Osaka City University Hospital, Osaka, Japan; 4 Data Science Center, Promotion Unit, Institute of Health Science Innovation for Medical Care, Hokkaido University Hospital, Sapporo, Japan

**Keywords:** Anxiety disorder, Aromatherapy, Sniffing lavender aroma, Study protocol, A multicenter, Double-masked, Randomized, Placebo-controlled clinical trial

## Abstract

**Background:**

Anxiety disorder is the most prevalent psychiatric disorder. Benzodiazepines, which are often used for anxiety in patients with anxiety disorder, have various side effects. Lavender, one of the most commonly used essential oils in aromatherapy, has the potential to reduce benzodiazepine use for anxiety disorders.

**Methods:**

This study is a multicenter, double-masked, randomized, placebo-controlled clinical trial. The study will recruit patients aged 20–59 years old with generalized anxiety disorder and panic disorder among anxiety disorders. The bottle containing the test solution (lavender aroma essential oil or distilled water) will be given to the patients. Patients will carry the bottles with them in their daily life and use the drops on tissue paper when anxious. The primary endpoint is the number of times anxiolytics used in 28 days.

**Discussion:**

If the use of benzodiazepines could be reduced by sniffing lavender aroma, which is inexpensive and safe, it would contribute not only to the risks associated with benzodiazepine use but also to the health care economy and could even be added as a standard treatment.

**Trial registration:**

University hospital Medical Information Network Clinical Trials Registry (UMIN-CTR), ID: UMIN000034422 Registered 17 January 2019.

**Supplementary Information:**

The online version contains supplementary material available at 10.1186/s12906-023-04231-1.

## Background

Anxiety disorder is an extremely common mental disorder [1], and its frequency has increased over the last decade [2]. A meta-analysis conducted by Lijster et al. showed that 21.3 years is the average age at the onset of anxiety disorders [3]. In the European Study of the Epidemiology of Mental Disorders, an international epidemiological study conducted in Europe (Belgium, France, Germany, Italy, the Netherlands, and Spain) among people aged 18 years and older, the lifetime prevalence of anxiety disorders was 13.6% [4]. The World Mental Health Japan Survey, conducted among Japanese residents aged 20–75, reported a 10.8% lifetime prevalence of anxiety disorders [5]. Anxiety disorders have been found to contribute to increased rates of absenteeism in both work and educational settings, resulting in a greater financial burden when compared to other mental diseases [6]. According to the fifth version of Diagnostic and Statistical Manual of Mental Disorders (DSM)-5, major anxiety disorders that affect adults include generalized anxiety disorder (GAD), panic disorder (PD), social anxiety disorder (SAD), agoraphobia, and specific phobia [7].

The most common treatment for anxiety disorders is a combination of pharmacotherapies, including the use of benzodiazepines [8], serotonin reuptake inhibitors (SSRIs), serotonin-norepinephrine reuptake inhibitors [9], and psychotherapy (cognitive behavioral therapy) [10, 11]. Usually, patients with anxiety disorders use benzodiazepines for transient anxiety symptoms such as panic attacks. However, their prolonged use may have adverse health effects despite their short-term benefits. These factors encompass the potential for dependence and adverse outcomes, such as cognitive decline [12, 13].

Aromatherapy is one of the most extensively used supplementary therapies [14]. It is based on the therapeutic application of essential oils produced from aromatic herbs that can be ingested, applied topically to the skin, or inhaled [15]. Some essential oils have been known as anxiolytic candidates, namely lavender, also known as *Lavandula angustifolia* Mill. A randomized placebo-controlled study of 116 volunteers showed reduced stress levels in the group using lavender oil for aromatherapy compared with the placebo group [16]. In adults with GAD, the orally administered anxiolytic effect was comparable to that of benzodiazepines [17] and SSRIs [18], and fewer adverse effects were reported than with synthetic drugs.

The aforementioned research may suggest the possibility of aromatherapy as a treatment for anxiety disorders. However, these do not include studies of inhaling or sniffing essential oil. While inhaling essential oils changes moods in healthy volunteers [19], a study on women undergoing abortions indicates that aromatherapy cannot be substituted for anxiolytics [20]. Therefore, anxiolytic effects of inhaling essential oils still need to be elucidated. Additionally, it should be noted that inhaling and sniffing may exhibit different effects because inhaling essential oil means the oil is gas while sniffing means the oil can be in various forms, such as liquid or solid.

In summary, to the best of our knowledge, the possibility of chronic exposure to sniffed essential oil as an aromatherapy for treating anxiety disorders has never been evaluated. Therefore, the effect of sniffing fragrance in aromatherapy should be elucidated first before delving into which essential oils show an anxiolytic effect.

In this study, starting with lavender as our initial focus, we will investigate the efficacy and safety of sniffing lavender aroma in patients with anxiety disorders, aiming to develop a reliable and safe alternative and complementary treatment.

## Design

### Study design

This study is a multicenter, double-blind, randomized, and placebo-controlled clinical trial. To compare the effectiveness of treatment with lavender aroma, we will compare two groups of patients: a lavender aroma group and a non-lavender aroma group who were provided with a placebo. Double masking is necessary due to the possibility of changes in medical treatment once the researcher knows the allocation results.

### Patients

The study will include patients aged 20–59 with GAD and PD among anxiety disorders diagnosed using the DSM-5. The study dates ranged between September 9, 2018, and March 31, 2024. Posters and other methods will be for study recruitment. We will explain everything using permission from the explanatory paper and consent forms, and we will obtain everyone’s signed consent. Written informed consent will be obtained from all the participants. Moreover, participants can withdraw at anytime after participating in this study.

#### Inclusion criteria

Patients must satisfy the following requirements:

1. Only female patients aged 20–59 who consented to the study.
2. Patients who were diagnosed with GAD or PD using the DSM-5.
3. Patients who have taken anxiolytics more than seven times within the past month.
4. Patients with no history of aroma use.

#### Basis for setting inclusion criteria

1. The patient’s age was defined as the age when the patient consented to the study. The effectiveness of the aroma may differ according to sex. To detect efficacy efficiently, the study needed to be limited to a more homogeneous population and was therefore limited to female patients only.
2. Patients with GAD and PD were chosen as they are most frequently encountered in daily practice.
3. We included patients who took anxiolytics because we expected these patients to benefit from the treatment.
4. Patients who had never used aroma-related products were chosen to eliminate the possibility that a history of aroma use influenced this study.

#### Exclusion criteria

Patients who fell into the following criteria below are excluded from the study.

1. Patients who are diagnosed with significant physical and mental health issues by the physician in charge.
2. Patients who are pregnant, possibly pregnant, within 28 days postpartum, or are breastfeeding.
3. Patients who are diagnosed with any olfactory dysfunction or chronic nasal obstruction.
4. Patients with dilated thoughts of death.
5. Patients using anxiolytics other than benzodiazepines as an abortive medication for anxiety attacks.
6. Any patients deemed ineligible by the principal investigator or sub-investigator for this study were excluded.

#### Basis for setting exclusion criteria

The above criteria in points 1, 4, and 6 were used to ensure the general safety of the patients.

Point 2 was established because the safety of the patient treatment for pregnant women, fetuses, and newborns was not established.

Points 3 and 5 were established to exclude patients who would be affected by the evaluation of the treatment under study.

### Interventions

**Fig. 1:**
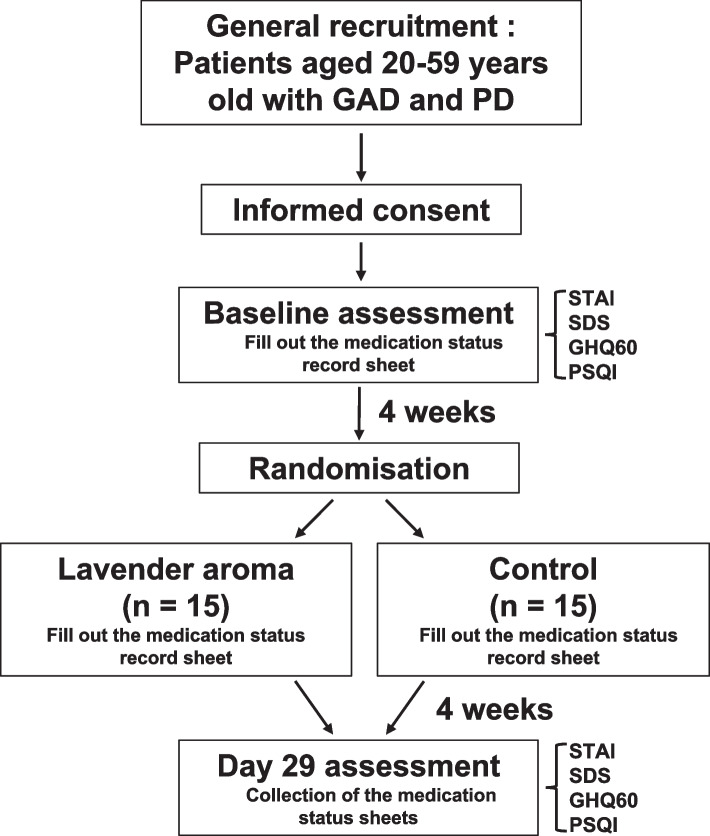
Flowchart of the study schedule. GAD = generalized anxiety disorder; PD = panic disorder; STAI = State Trait Anxiety Inventory; SDS = Self-Rating Depression Scale; GHQ60 = General Health Questionnaire 60; PSQI = Pittsburgh Sleep Quality Index

The study schedule is depicted in Fig. 1. After obtaining the 4-week baseline benzodiazepine medication status and other information, the patients will be assigned to either the aromatherapy or distilled water (placebo) groups. The patient will begin carrying the bottle immediately after the outpatient visit, and in this study, carrying is defined as carrying the bottle at all times in a bag or similar while going out. However, for the time spent at home, the patient is considered to be carrying the bottle because the test solution is always available if the patient experienced anxiety attacks.

Lavender aroma (*L. angustifolia* ssp. Angustifolia (Pranarom International, Ghislenghien, Belgium) or distilled water is used as the test solution, which is stored in a 10 ml glass bottle with a drop stopper (Pranarom International). The bottle containing the test solution (lavender aroma essential oil or distilled water) will be given to the patient without informing them of the type of test solution. Patients will be asked to carry the bottles with them daily at all times and use the drops on the tissue paper when anxious. The patients will be instructed to maintain a diary in which they will record details on their benzodiazepine medication and aroma usage (Supplementary Fig. 1).

### Data management

The study data will be collected and stored in the Research Electronic Data Capture system (REDCap), a web-based, secure, Health Insurance Portability and Accountability Act-compliant research management platform [21, 22], which is also an electronic data capture system for electronically gathering clinical data in clinical research. This system provides comprehensive support for various research procedures, encompassing patient scheduling, patient randomization, data entry and management, data safety monitoring, and adverse event reporting. One of the main advantages of REDCap is that it requires a thorough audit trial. The individual providing the input is also immediately alerted if any field is blank or values outside the acceptable range are entered. Backup paper copies were given when internet connectivity was lost, and these copies were then entered into REDCap.

The study database will not contain any personal identifiers, such as names or other information that might potentially lead to the direct identification of a patient by a third party; instead, the enrolled patients’ data were accessed and queried using the REDCap software. To prevent unauthorized access, the datasets saved on computers are password-protected and encrypted. Patients will be identified by numbers or symbols, and the relationship between these and details that may be used to quickly identify a person (such as name or address) to a third party will not be maintained during data collection. It is necessary to maintain a record of the correspondence between information that is clearly identifiable to a third party (such as name and address) in a locked cabinet (personal information manager: HA).

### Primary endpoint

The primary outcome measure is the number of times anxiolytics were used within 28 days.

### Secondary endpoint

1. Total amount of anxiolytic used (diazepam equivalent)
2. Change in State Trait Anxiety Inventory (STAI) total score from baseline at 28 days.
  2.1. Change from baseline in state anxiety total score at 28 days.
  2.2. Change from baseline in trait anxiety total score at 28 days.
  The STAI is a 40-item self-report scale that assesses the presence and intensity of anxiety symptoms as well as the generalized tendency to be anxious [23]. By asking patients how they feel “right now”, the State Anxiety Scale assessed their present level of anxiety, including their subjective emotions of tension, uneasiness, concern, and activation of their autonomic nervous system. There are 20 elements in total, with values ranging from 0–2. The Trait Anxiety Scale measures characteristics of anxiety propensity that are generally constant, such as overall tranquility, assurance, and security [24]. There were 20 items in total, with values ranging from 0–2. Higher ratings indicate severe anxiety.
3. Change in Self-Rating Depression Scale (SDS) total score from baseline at 28 days.
  The SDS, which includes 20 items on sadness, crying easily, and sleep disorders, is used to assess patients’ depression and changes in therapeutic processes [25]. The scoring system adopts a 4-level scoring system, among which 10 items are positive scoring and the other 10 items are negative scoring. A score < 53 indicates normal, a score 53–72 indicates depression, and a score > 72 indicates major depression. The severity of the depression increases with score.
4. Change in the General Health Questionnaire 60 (GHQ60) total score from baseline at 28 days.
  The GHQ60 is a 60-item self-rating scale with a range of 0–60 that assesses physical and mental suffering and the likelihood of developing psychiatric illnesses. A high risk of neurosis is indicated by a score of 17 or above [26].
5. Change in the Pittsburgh Sleep Quality Index (PSQI) total score from baseline at 28 days.
  A self-rated questionnaire called the PSQI rates a month’s worth of sleep disruption and quality. The seven “component” scores, namely subjective sleep quality, sleep latency, sleep length, habitual sleep efficiency, sleep disturbances, utilization of sleeping drugs, and daytime dysfunction, are derived from the analysis of the 19 individual items. The cumulative score is derived from the summation of the points attributed to each of the seven constituent components. An overall score of 5 or above is indicative of poor sleep quality [27].
6. Number of anxiety attacks.

### Sample size calculation

Among the patients treated by the principal investigators and research assistants at the facilities where this study was conducted, those with anxiety disorders suitable for the study generally used anxiolytic medications 30 times/month. We expected the use of lavender aroma would decrease this frequency to about 10 times/month and assumed that the difference in the mean frequency between the distilled water group and the lavender aroma group would be -10 ± 7.5 times/month. If a two-sample t-test is conducted with a two-sided significance level of 5% and power of 80%, 10 patients per group are required. The total sample size is 30 (15 per group), considering that discontinuation or dropout occurs approximately 30% of the time.

### Randomization

Four weeks after the start date, the patients will be allocated to two groups in a random manner, employing a stratified permuted block approach (stratification factors are the primary illness (GAD or PD)) on REDCap. The results of the assignment will be indicated by a number, and a bottle containing the test solution (lavender aroma essential oil or distilled water) will be provided by the Department of Orthodontics, Kagoshima University Graduate School of Dentistry, where the bottles containing the test solution matching the number will be kept without informing the investigators or patients of the type of test solution.

### Statistical analysis

#### Analysis set

For the next analysis sets, statistical analysis will be done.

1. Full analysis set (FAS)
  All patients will be regarded as FAS, with the exception of those who received care in flagrant violation of ethical standards, those who were not given the test solution, and those whose data for the key outcome were missing.
2. Safety analysis set (SAS)
  The SAS will be referred to as the group of patients to whom the test solution was distributed.
3. Per protocol set (PPS)
  The PPS will be referred to as the group of patients who fall under the FAS but do not match the criteria below.
  3-1. Patients who fail to satisfy the predetermined inclusion criteria or contravene the established exclusion criteria for this study
  3-2. Notable departures from the established study procedure
  3-3. Patients who had not carried a bottle containing the test solution for more than 20 days

#### Summary between treatment groups

For each FAS, patient backgrounds will be tallied by therapy group. For continuous data, summary statistics will be provided, and for categorical data, frequencies and proportions (%) will be shown.

#### Primary endpoint

The primary endpoint analysis will be based on the FAS. A sensitivity analysis will be conducted on the PPS to confirm the robustness of the results. This will be analysed using an analysis of covariance model for the change from the baseline to 28 days in the number of times anxiolytic medication is used, with treatment and the baseline value as covariates.

In case of discontinuation, dropout, the number of times anxiolytics per period will be converted to 28 days for the analysis. Missing values will not be imputed.

#### Secondary endpoints

Secondary endpoints will be analysed in the same way as that for the primary endpoint.

All statistical analyses will be performed with SAS software (version 9.4; SAS Institute, Cary, NC, USA).

#### Interim analyses

No interim analysis is planned.

### Monitoring

To ensure that this study is being carried out properly, the lead investigator will request that the institution perform monitoring. As a usual practice, the monitoring report will be submitted annually.

### Adverse events

The incidence of adverse events is minimal, and no discernible health or safety concerns have been reported in relation to the participants included in the clinical research. The safety monitoring group will oversee data monitoring. Throughout the course of the 1-week program, any small or significant occurrences related to the intervention or routine care groups will be observed. The chief investigator will review any negative or unexpected consequences.

## Discussion

The objective of this study is to examine the effectiveness and safety of olfactory exposure to essential oil fragrance among individuals diagnosed with anxiety disorders, with the aim of establishing a dependable and secure alternative and adjunctive therapeutic approach for anxiety management in this population. Lavender was taken as a starting point for exploring the use of essential oil fragrances as potential anxiolytic treatments, because a systematic review of aromatherapy on anxiety clearly indicates that it is the major fragrance [28]. Considering this objective, the placebo should meet the following criteria: 1) Be indiscernible from its appearance, i.e., a colorless clear liquid; 2) Having no fragrances. Therefore, our placebo of distilled water is justified. In fact, a systematic review of lavender as a potential anxiolytic supports that using distilled water as a placebo is appropriate [29].

However, there are some limitations to be considered. First, it should be noted that secondary endpoints of STAI, SDS, GHQ60, and PSQI are subjective. Therefore, patients’ objective psychological states or sleep qualities, such as functional magnetic resonance imaging, heart rate variability, or polysomnography might strengthen our findings. Because these were not the primary endpoint, we do not perform further objective assessment. Second, the primary endpoint of the number of times anxiolytics used is not completely objective. Although the possibility may not be high, we should be cautious about the accuracy of patients’ reporting of their anxiolytics use.

Last but not least, limitations of statistical analyses should be seriously discussed. The difference of primary endpoint in the mean frequency of benzodiazepine use between the distilled water group and the lavender aroma group of -10 ± 7.5 times/month was derived from a simple assumption. Therefore, we cannot deny the possibility of manipulating the sample size. Nonetheless, this assumption was based on our clinical experience of patients with mood disorders by the intervention of complementary medicine, such as mindfulness. Another statistical limitation lies in imputation. If missing values tend to appear in one common characteristic of patients, using imputation to handle such patients could reveal significant insights. However, because the sample size was calculated considering dropout rate of 30%, and such analysis can be performed separately, we decided to perform no imputation.

Considering aforementioned limitations, if the use of benzodiazepines could be reduced by sniffing inexpensive and safe essential oils, including lavender, this would contribute not only to the risks associated with benzodiazepine drug use, but also to the health care economy. In addition, since sniffing aromas is simpler than taking medications with drinking water, the use adherence is likely to be higher and it could be added as a standard treatment.

### Supplementary Information

**Additional file 1: Supplementary Figure 1.** Diary of use of test solution and anxiolytics.

## Data Availability

Not applicable.

## Abbreviations

AMED: Japan Agency for Medical Research and Development
FAS: Full analysis set
GAD: Generalized anxiety disorder
GHQ60: General Health Questionnaire 60
PD: Panic disorder
PPS: Per protocol set
PSQI: Pittsburgh Sleep Quality Index
SAD: Social anxiety disorder
SAS: Safety analysis set
SSRIs: Serotonin reuptake inhibitor
STAI: Stait Trait Anxiety Inventory
REDCap: Research electronic data capture system
