# Supplementary material for: The effect of lavender aroma for anxiety disorder: a study protocol for a multicenter, double-masked, randomized, placebo-controlled clinical trial
Source: BMC Complement Med Ther. 2023 Nov 6;23:397. doi: 10.1186/s12906-023-04231-1 (PMC10626714; doi:10.1186/s12906-023-04231-1)
Supplement: Supplementary file 1 — Additional file 1: Supplementary Figure 1. Diary of use of test solution and anxiolytics. [file 12906_2023_4231_MOESM1_ESM.pptx]

## Slide 1
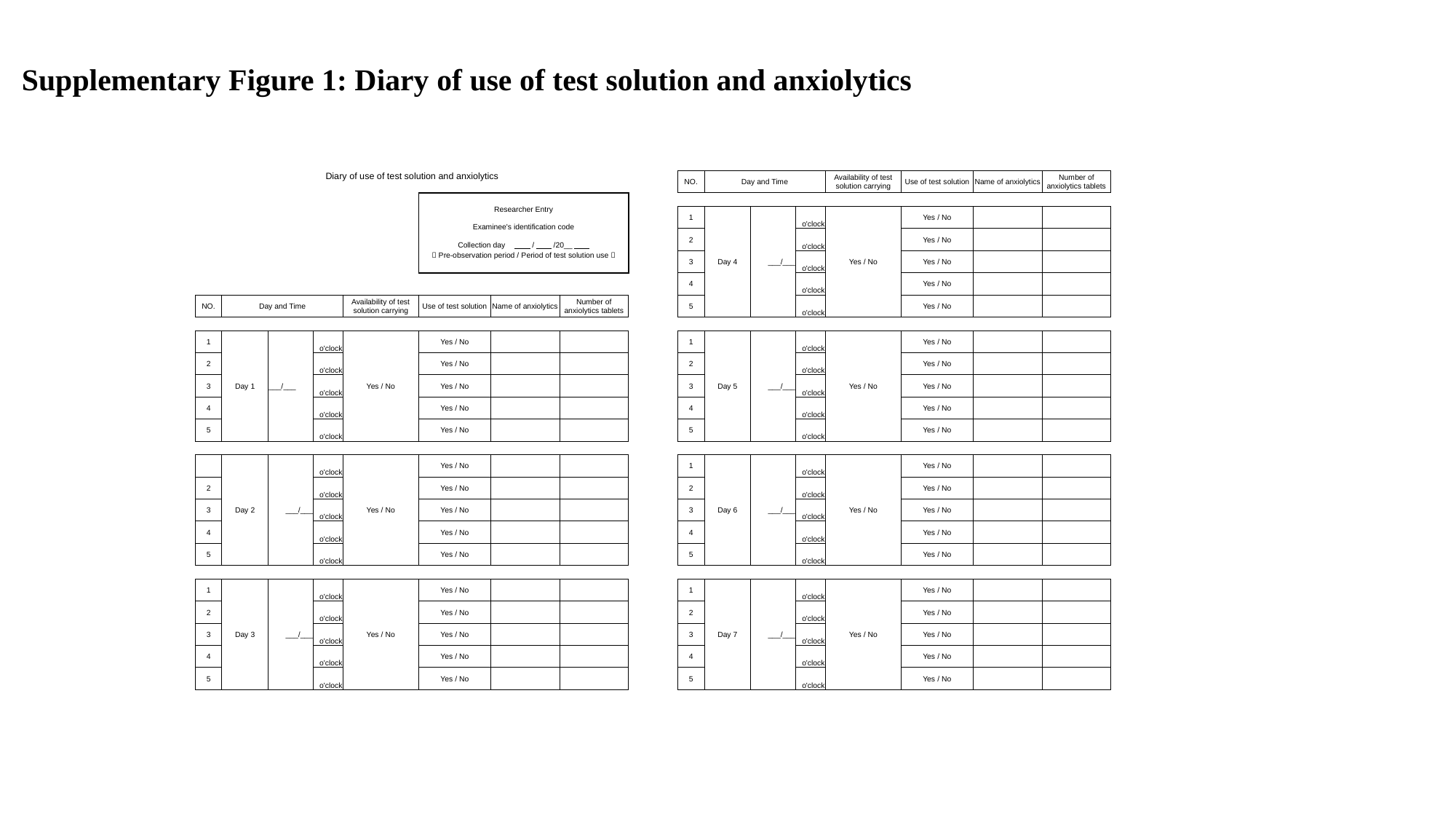

Supplementary Figure 1: Diary of use of test solution and anxiolytics
| Diary of use of test solution and anxiolytics | | | | | | | | | NO. | Day and Time | | | Availability of test solution carrying | Use of test solution | Name of anxiolytics | Number of anxiolytics tablets | |
| --- | --- | --- | --- | --- | --- | --- | --- | --- | --- | --- | --- | --- | --- | --- | --- | --- | --- |
| | | | | | Researcher Entry Examinee's identification code Collection day　　　/　　/20\_\_　　 （Pre-observation period / Period of test solution use） | | | | | | | | | | | | |
| | | | | | | | | | 1 | Day 4 | \_\_\_/\_\_\_ | o'clock | Yes / No | Yes / No | | | |
| | | | | | | | | | 2 | | | o'clock | | Yes / No | | | |
| | | | | | | | | | 3 | | | o'clock | | Yes / No | | | |
| | | | | | | | | | 4 | | | o'clock | | Yes / No | | | |
| NO. | Day and Time | | | Availability of test solution carrying | Use of test solution | Name of anxiolytics | Number of anxiolytics tablets | | 5 | | | o'clock | | Yes / No | | | |
| | | | | | | | | | | | | | | | | | |
| 1 | Day 1 | \_\_\_/\_\_\_ | o'clock | Yes / No | Yes / No | | | | 1 | Day 5 | \_\_\_/\_\_\_ | o'clock | Yes / No | Yes / No | | | |
| 2 | | | o'clock | | Yes / No | | | | 2 | | | o'clock | | Yes / No | | | |
| 3 | | | o'clock | | Yes / No | | | | 3 | | | o'clock | | Yes / No | | | |
| 4 | | | o'clock | | Yes / No | | | | 4 | | | o'clock | | Yes / No | | | |
| 5 | | | o'clock | | Yes / No | | | | 5 | | | o'clock | | Yes / No | | | |
| | | | | | | | | | | | | | | | | | |
| | Day 2 | \_\_\_/\_\_\_ | o'clock | Yes / No | Yes / No | | | | 1 | Day 6 | \_\_\_/\_\_\_ | o'clock | Yes / No | Yes / No | | | |
| 2 | | | o'clock | | Yes / No | | | | 2 | | | o'clock | | Yes / No | | | |
| 3 | | | o'clock | | Yes / No | | | | 3 | | | o'clock | | Yes / No | | | |
| 4 | | | o'clock | | Yes / No | | | | 4 | | | o'clock | | Yes / No | | | |
| 5 | | | o'clock | | Yes / No | | | | 5 | | | o'clock | | Yes / No | | | |
| | | | | | | | | | | | | | | | | | |
| 1 | Day 3 | \_\_\_/\_\_\_ | o'clock | Yes / No | Yes / No | | | | 1 | Day 7 | \_\_\_/\_\_\_ | o'clock | Yes / No | Yes / No | | | |
| 2 | | | o'clock | | Yes / No | | | | 2 | | | o'clock | | Yes / No | | | |
| 3 | | | o'clock | | Yes / No | | | | 3 | | | o'clock | | Yes / No | | | |
| 4 | | | o'clock | | Yes / No | | | | 4 | | | o'clock | | Yes / No | | | |
| 5 | | | o'clock | | Yes / No | | | | 5 | | | o'clock | | Yes / No | | | |
